# In-TFT-Array-Process Micro Defect Inspection Using Nonlinear Principal Component Analysis

**DOI:** 10.3390/ijms10104498

**Published:** 2009-11-20

**Authors:** Yi-Hung Liu, Chi-Kai Wang, Yung Ting, Wei-Zhi Lin, Zhi-Hao Kang, Ching-Shun Chen, Jih-Shang Hwang

**Affiliations:** 1 Department of Mechanical Engineering, Chung Yuan Christian University, Chungli, 320, Taiwan; E-Mails: winroger@yahoo.com.tw (C.-K.W.); yung@cycu.edu.tw (Y.T.); levi5382001@hotmail.com (W.-Z.L.); kzh7103@hotmail.com (Z.-H.K.); 2 Mechanical and Systems Research Laboratories, Industrial Technology Research Institute, Hsinchu 310, Taiwan; E-Mail: cschen6922@itri.org.tw (C.-S.C.); 3 Institute of Optoelectronic Sciences, National Taiwan Ocean University, Keelung, 202, Taiwan; E-Mail: hjsspin@mail.ntou.edu.tw (J.-S.H.)

**Keywords:** thin film transistor liquid crystal display, TFT array process, automatic optical inspection, defect inspection, kernel principal component analysis, support vector machine

## Abstract

Defect inspection plays a critical role in thin film transistor liquid crystal display (TFT-LCD) manufacture, and has received much attention in the field of automatic optical inspection (AOI). Previously, most focus was put on the problems of macro-scale Mura-defect detection in cell process, but it has recently been found that the defects which substantially influence the yield rate of LCD panels are actually those in the TFT array process, which is the first process in TFT-LCD manufacturing. Defect inspection in TFT array process is therefore considered a difficult task. This paper presents a novel inspection scheme based on kernel principal component analysis (KPCA) algorithm, which is a nonlinear version of the well-known PCA algorithm. The inspection scheme can not only detect the defects from the images captured from the surface of LCD panels, but also recognize the types of the detected defects automatically. Results, based on real images provided by a LCD manufacturer in Taiwan, indicate that the KPCA-based defect inspection scheme is able to achieve a defect detection rate of over 99% and a high defect classification rate of over 96% when the imbalanced support vector machine (ISVM) with 2-norm soft margin is employed as the classifier. More importantly, the inspection time is less than 1 s per input image.

## 1. Introduction

Over the past decade the TFT-LCD has been a popular flat panel display choice. With the increase in demand, the sizes of the TFT-LCDs have been getting larger. To make large-sized TFT-LCDs, the original manufacturing process needs to be changed to meet the requirements. Such a change usually results in various defects, which decrease the yield rate significantly, therefore, defect inspection plays a key role in TFT-LCD manufacture. However, in current practice, this task still relies heavily on human observers, which is not only time consuming, but also prompt to be unreliable. Accordingly, automatic optical inspection (AOI) has been suggested as the most efficient way to detect defects.

TFT-LCD manufacture consists of three processes, namely the TFT array process, the cell process, and the module assembly process. In recent years, there has been a large body of work regarding the so-called mura-defect detection, *e.g.*, [1–4]. Mura is a serious kind of defect and needs to be detected in the cell process. Once a mura defect is found in a panel, this panel must be discarded if not repairable, which raises the production costs greatly. In fact, most mura defects are caused by the inline defects of the TFT array process. The inline defects vary greatly, and their sizes are too small to be observed, making the problem of inline defect inspection intractable.

Inline defect inspection involves three sub-tasks: defect detection, target defect identification, and classification. Defect detection refers to judging whether an image contains a defect or not, and target defect identification means determining whether the defect detected is crucial to the product yield. Defect classification plays a critical role in production-equipment diagnosis because different defects have different causes. Liu *et al.* [5] have recently proposed a system to deal with the problem of inline defect detection, which was developed based on the locally linear embedding (LLE) method [6] and the support vector data description (SVDD) [7]. LLE is a manifold learning method to extract nonlinear features from a pattern. However, it suffers from the *out-of-sample* problem [8,9]. SVDD is essentially a one-class classifier. Although it is efficient for anomaly detection, it cannot be applied in a multi-class classification problem. For inline defect classification, the SVDD is not a good candidate. Liu *et al.* [10] have also proposed a target defect identification system. In their work the SVDD was extended to an SVDD ensemble for modeling the target defects. If a test pattern is accepted by the SVDD ensemble, the pattern belongs to the target class. In TFT array process, the target defects would cause serious damages to the LCD panels.

According to the above analysis, it is known that the third sub-task is still an issue to be solved. In this paper we present a new inline-defect inspection scheme that not only possesses the functions of defect detection and target defect identification, but can also simultaneously accomplish the task of defect classification.

Inline defect classification is typically a pattern recognition problem. In particular, inline defect patterns suffer from large variations in shape, color, texture, and size. Therefore the two issues, how to provide an effective representation method and how to design a classifier with high generalization performance, are the keys to acheiving high defect detection accuracy.

Principal component analysis (PCA) is a popular subspace analysis method for pattern representation and reconstruction. However, due to its linear nature [11], its performance is sometimes limited. Recently, a nonlinear version of PCA has been proposed, called kernel PCA (KPCA) [12]. KPCA first maps the input data into a higher dimensional feature space via a nonlinear mapping, then performs the linear PCA in that space to find a set of eigenvectors that are nonlinearly related to the input data. Thus, KPCA can capture the nonlinear relationships between pixels in an image, and extract more discriminating features from an image and reduce the dimensionality of the input image. In face recognition studies, *e.g.*, [13], KPCA has shown to have better performance than PCA in terms of feature extraction. To enhance the LCD defect detection/classification rate, in this paper we adopt the powerful KPCA as the feature extractor.

Aside from feature extraction, classifier design is also crucial to defect inspection. In the fields of pattern recognition and machine learning, support vector machine (SVM) has received much attention over the past decade. The learning strategy of SVM is based on the principle of structural risk minimization [14], so SVM has better generalization ability than other traditional learning machines that are based on the learning principle of empirical risk minimization, such as multi-layer neural networks trained by error back-propagation algorithm [15,16]. Thus, the SVM is a good candidate for our work. However, in practice, using the SVM as the defect classifier may not achieve the optimum classification performance due to the nature of the problem, explained as follows.

In TFT array process, various kinds of inline defects would occur. Their occurrence frequencies are different. For example, “particle” is the most commonly-seen defect while the defect “abnormal photo-resist coating” seldom appears. It implies that the available training samples for each defect would be different, leading to a very imbalanced training dataset. In SVM, the error penalties for positive and negative classes are the same. This will make the learned optimal separating hyperplane (OSH) move toward the smaller class. More precisely, if the positive class is smaller than the negative class, then the OSH will move toward the positive class, which will further result in numerous false negative errors. We call this phenomenon the “class-boundary-skew (CBS) problem”. Due to this problem, the success of using SVM in defect detection and classification is limited. Therefore, how to solve the CBS problem when applying SVM to defect inspection becomes a very critical studying issue.

Several works have proposed ways to solve the CBS problem [17–22]. The methods of [20,21] use different sampling techniques to the training data before data enter the classifier. The different error cost (DEC) algorithm of [17,19] is embedded into the formulation of SVM such that the skew phenomenon of the OSH can be corrected. This method does not change the information of the data structure beforehand. The SDC method [18] combines the SMOTE [22] and the different error cost algorithm [17]. For LCD defect inspection, since every defect image stands for one particular defect information, we do not intend to use any pre-sampling techniques like those fall into the first category that may change the data structure. Therefore, the DEC algorithm [17] is adopted in this paper to deal with the CBS problem due to the imbalanced defect training dataset. By introducing the DEC algorithm to SVM, the imbalanced SVM (ISVM), a variant of SVM is proposed. In fact, the concept of ISVM is similar to that of adaptive SVM proposed in [23]. However, in their work only 1-norm soft margin is considered. In this paper, we reformulate the ISVM with 2-norm soft margin, and provide the corresponding KKT conditions. Results will show that the proposed version of 2-norm soft margin ISVM achieves better defect classification performance.

## 2. Results and Discussion

In this section we introduce the image acquisition in a real TFT array process, as well as the types of the inline defects. After that, we illustrate the proposed inline defect inspection scheme. The KPCA is also introduced, and the ISVM with 2-norm soft margin will be reformulated in the end of this section.

### 2.1. Image Acquisition and Defect Analysis

**Figure 1.**
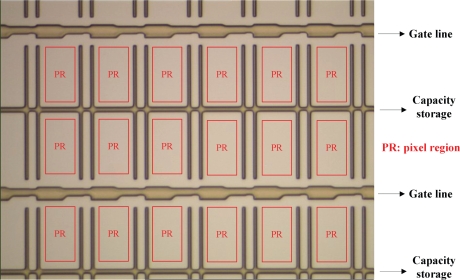
An example of normal GE image. A normal GE pattern consists of three parts. The first part is the GE lines, which are the thicker lines appearing in the pattern periodically. The maximum width of a GE line is around 15 micro meters. The second part is the capacity storages (CSs), which are thinner than the GE lines. The third part is the rectangular regions surrounded by GE lines and CSs, called pixel regions (PRs).

TFT-LCD manufacture is composed of three processes: the TFT array, cell and module assembly processes. TFT array process consists of five successive engineering steps, including gate electrode (GE), semiconductor electrode (SE), source and drain (SD), contact hole (CH), and pixel electrode (PE) engineering, each of which would generate a distinct pattern on the glass substrate. Each of the five engineering steps contains the same five processes, including cleaning, thin film deposition, photolithography (which contains three sub-processes: photoresist coating, exposure, and developing), etching, and stripping. In this paper we focus on the first engineering of TFT array process, the GE engineering, because the earlier the inline defects are detected and classified, the earlier the defective LCD panels can be repaired. A normal GE image is shown in Figure 1.

**Figure 2.**
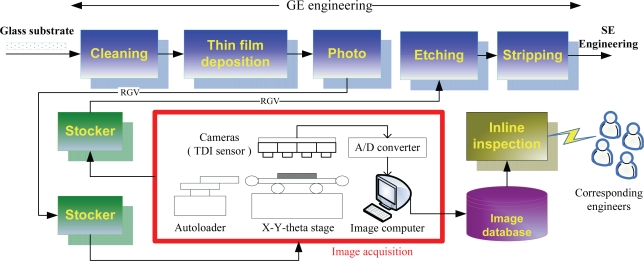
The flow chart of the image acquisition process in real GE engineering.

To inspect defects in real time, we placed a test bed in the plant to acquire the surface images of the LCD panels. Notice that the images should be captured before the etching process, because the defective panels can still be repaired by rework as long as they have not yet been sent into the etching process. The image acquisition process is introduced as follows, and is depicted in Figure 2.

After a sheet of glass substrate containing 6 LCD panels completes the photoprocess, it will be carried to the stocker by a rail-guided vehicle (RGV). At the stocker, a cassette containing 25 sheets of glass substrates is carried to the inspection equipment. After the cassette arrives, the inspection equipment will start to pick six out of the 25 sheets randomly, and each of the six chosen substrates will be put on an X-Y-theta stage by an autoloader, one at a time. Above the stage, there are four TDI (Timing Delay Integration) line-scan cameras placed on the inspection equipment.

Once a sheet of glass substrate is placed on the stage, the cameras begin to scan its surface. During the scanning, the areas on the surface to be scanned are randomly determined, and the number of areas to be scanned is determined by the user. There are 30 areas in the present case. The scanned analog signals are transferred to digital signals (images) *via* an analog-to-digital (A/D) converter. Therefore, totally 30 images will be captured from the surface of a substrate. These images will be stored in the image computer temporarily. Usually, it would take around four minutes to scan a sheet of glass substrate. After the six glass substrates are scanned, all the 180 acquired digital images will be stored in an image database. Each image is a 768× 576 24-bit colored image (JPEG format), and has the resolution of around 1.15–1.20 (pixels/μm). Finally, the cassette will be carried back to the stocker, and sent to the next process, *i.e.*, the etching process.

**Figure 3.**
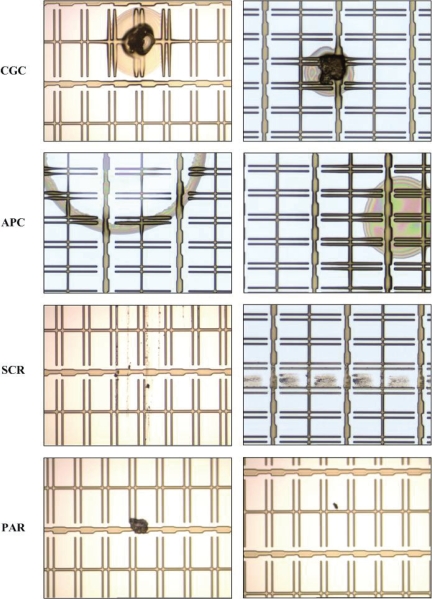
Examples of the four types of inline defects.

Four kinds of inline defects are common in GE engineering: *connection between GE and CS* (CGC), *abnormal photo resist coating* (APC), *scratch* (SCR), and *particle* (PAR). The causes of occurrence of the four kinds of inline defects are listed in Table 1, and some examples of inline defects are shown in Figure 3.

Among the four kinds of inline defects, the first three (CGC, APC, and SCR) are critical because they would cause different types of mura defects on the panels. For example, SCR would cause the line-type mura defect. Once a panel is found with a mura defect, it is generally be discarded. Therefore, the first three are considered as the target defects. As for PAR defect, it is consider as the non-target defect because particles can be removed by cleaning before the LCD panels enter the etching process Note that the particles mean the ones falling on the panels after the photo process is complete.

### 2.2. Inline Defect Inspection Scheme

**Figure 4.**
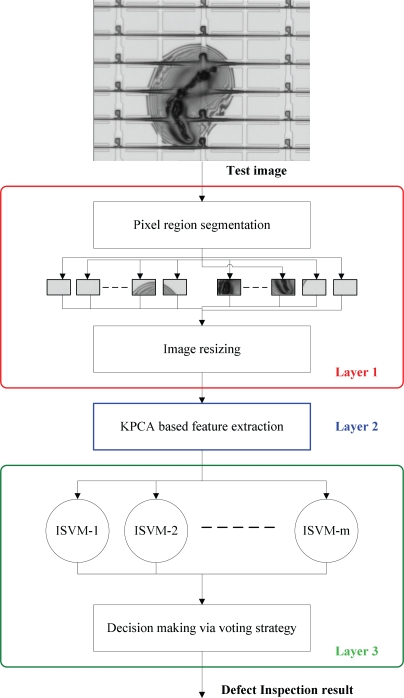
The flow chart of the inline defect inspection scheme.

Figure 4 depicts the flow chart of the inline defect inspection scheme, which is composed of three layers, the image preprocess layer (layer 1), the feature extraction layer (layer 2), and the defect classification layer (layer 3). In the following we explain this scheme in more details.

#### 2.2.1. Layer 1: Image Preprocessing

From Figure 3, it can be observed that the defect in a defective image would appear in some PRs, called the defective PRs, while other PRs in the same image are normal. The main difference between a normal PR and a defective one is that their appearances are apparently different: the surface of a defective PR contains a specific kind of texture, while the gray-level distribution of a normal pixel is nearly uniform. Therefore, the appearance of a PR is a discriminating feature. The goal of the proposed scheme is to judge whether a PR is normal or not, and to further recognize the type of the defect if the PR is defective.

Firstly, after receiving a test image, the PRs are automatically segmented from the input gray-level image by a projection-based PR segmentation method proposed in [5]. Then, the segmented PRs are resized to have the same 50 × 50 size. After row-by-row scanning, each PR image can be represented by a vector of 2,500 × 1, in which the elements are the gray-level values within [0,255]. Finally, the PR vectors are sent into the KPCA for further feature extraction, one at a time. In the following, a PR vector is called a PR datum.

#### 2.2.2. Layer 2: KPCA-Based Feature Extraction

Before using the KPCA to extract features from a PR pattern, we need to train the KPCA in advance.

##### Training

Suppose that we have the PR training set {*x_i_* ∈ *R^n^*}*_i_*_=1,...,_ *_M_* (*n =*2500). The idea of KPCA is to map the input data *x* into a high-dimensional feature space *F* via a nonlinear mapping function *φ* and then perform PCA in *F*. Assume that the training data are centered to have a zero mean distribution in *F*, *i.e.*, $\sum_{i=1}^{M}\varphi (x_{i})=0$ (the centering method of data in *F* can be found in Appendix B of [12]). KPCA aims to diagonalize the estimate of the covariance matrix of the mapped data *φ* (*x_i_*):

(1) $$\Gamma =\frac{1}{M}\sum_{i=1}^{M}\varphi (x_{1})\varphi^{T}\;(x_{i})$$

This is equivalent to solving the eigenvalue problem: *λ v* = Γ*v*, and finding eigenvectors *v* ∈ *F* associated with nonzero eigenvalues *λ*. By substituting (1) into the eigenvalue problem we have:

(2) $$\Gamma v=\frac{1}{M}\sum_{i=1}^{M}(\varphi (x_{i})\cdot v)\varphi (x_{i}).$$

Since all solutions *v* with *λ* ≠ 0 lie within the span of *φ* (*x_i_*),*i* = 1,..,*M*, there must exist the coefficients *a_i_*,*i* = 1,...,*M* such that

(3) $$v=\sum_{i=1}^{M}a_{i}\varphi (x_{i}).$$

Then the following equations are considered:

(4) $$\lambda (\varphi (x_{i})\cdot v)=(\varphi (x_{i})\cdot \Gamma v)\;\;\;\forall i=1,\ldots ,M.$$

Substituting (1) and (3) into (4) and defining a *M* × *M* kernel matrix *K: K_ij_* ≡ *k*(*x_i_*, *x_j_*) = (*φ* (*x_i_*) ·*φ* (*x_j_*)), where *k*(*x_i_*, *x_j_*) denotes the kernel function, then solving the eigenvalue problem *λ v* = Γ*v* is equivalent to solving:

(5) Mλa=Ka

for nonzero eigenvalues *λ_l_* and eigenvectors $a^{l}={\left(a_{1}^{l},\ldots ,a_{M}^{l}\right)}^{T}$ subject to the normalization condition λ*_l_* (*a^l^ a^l^*) =1.

##### Feature extraction

For the purposes of dimensionality reduction and feature extraction, the *d* eigenvectors associated with the first *d* largest nonzero eigenvalues are chosen as the projection axes such that *d* ≪ *n* and *d* ≪ *M*. Let *x* be a test PR datum, then its projection onto the *k-*th eigenvector *v^k^* in *F* is computed by:

(6) $$z^{k}=v^{k}\cdot \varphi (x)=\sum_{i=1}^{M}a_{i}^{k}(\varphi (x_{i})\cdot \varphi (x))=\sum_{i=1}^{M}a_{i}^{k}k(x_{i},x),$$

where *z^k^* is called its nonlinear principal components corresponding to *φ*. Since *k*=1,…,*d*, we can obtain *d* nonlinear principal components and they form a feature vector *z* = [*z*^1^, *z*^2^,..., *z^d^*]*^T^*. The optimal number of principal components needs to be determined experimentally. Finally, after extracting the KPCA features, the PR features vectors are sent into Layer 3 for further classification, one at a time.

#### 2.2.3. Layer 3: Defect Inspection via ISVMs

##### Multi-class ISVMs for defect classification

In this work, there are four kinds of inline defects to be classified. However, a normal PR may also enter the classifier. Therefore, there should be five classes to be classified; they are “CGC”, “APC”, “SCR”, “PAR” and “normality”, respectively. However, since SVM is a binary classifier, ISVM is also a binary classifier. Hence we need to extend the two-class ISVM to multi-class ISVMs. To achieve this, the one-against-one strategy suggested by [24] is adopted in this work. By using the one-against-one strategy, then Layer 3 consists of *m* = 5 × (5–1)/2 = 10 parallel ISVMs (see Figure 4). Each of the *m* ISVMs receives the same input *z*. After the classification with the input *z*, each ISVM will output its decision DK, −1 or +1. Finally, the voting strategy [24] aggregates the decisions, D1, D2, …, D*m*, and then outputs the final result, *i.e.*, the defect inspection result. More precisely, supposing that the *i*th ISVM (*i* = 1,…,10) is responsible for the classification of class *k* and class *k* ′ (*k, k′* = 1,…,5; *k′ ≠ k*), the voting strategy means that if the *i*th ISVM says that the input data *z* belongs to the *k*th class, then the vote for the *k*th class is added by one. Otherwise, the *k′*th class is added by one. Finally, *z* belongs to the class getting the most votes.

For an input *z*, if it is classified as the class “normality”, then the PR is a normal one. If it is classified as the class “PAR”, then the PR contains a particle. We can ignore this result because, as aforementioned, a particle is a non-target defect. However, if *z* is classified into one of the three categories: CGC, APC, and SCR, then the system will output the type of the defect to the corresponding engineers in the dust-free room *via* intranet (see Figure 2). Thus, the corresponding engineers can repair the defective PR in real time. In addition, the engineers can also diagnose and maintain the production equipment according to the type of the defect. For example, if an APC defect is found, then the photo-resist coating machine needs to be checked. It prevents the following LCD panels from suffering the same problem, thus being able to reducing the production cost and improving the yield rate significantly.

According to the mentioned above, it can be seen that the proposed inline inspection scheme can not only execute the functions of detect detection and target defect identification, but also accomplish the task of defect classification. Next, we reformulate the ISVM.

##### ISVM

Before introducing ISVM, we first review the SVM. Given the training set *S* = {*z_i_*, *y_i_*}, *i* = 1,..., *L*, where *z_i_* ∈ *R^d^* is the training data, and *y_i_* is its class label being either +1 or −1, let the weight vector and the bias of the separating hyperplane be *w* and *b*, the objective of SVM is to maximize the margin of separation and minimize the errors in the feature space, which is formulated as:

(7) $$\begin{array}{l} \text{Minimize}\;\frac{1}{2}{\left|\left|w\right|\right|}^{2}+C\sum_{i=1}^{L}\xi_{i}\\ \text{Subject}\;\text{to}\;\;y_{i}\left(w^{T}\varphi (z_{i})+b\right)-1+\xi_{i}\geq 0,\;\;\;\;\;\;\xi_{i}\geq 0,\;\;\;\forall i \end{array}$$

where *ξ_i_* are slack variables representing the error measures of data. The error weight *C* is a free parameter; it measures the size of the penalties assigned to the errors.

Form (7), it can be seen that in SVM, the error penalties the two classes are the same. The DEC algorithm [17] suggests that the cost of misclassifying a point from the small class should be heavier than the cost for errors on the large class. The basic idea is to introduce different error weights *C*^+^ and *C*^−^ for the positive and the negative class. If the positive class is larger than the negative class, then set *C*^+^ < *C*^−^; otherwise *C*^+^ > *C*^−^, which induces a decision boundary which is more distant from the smaller class. Accordingly, the ISVM can be formulated as follows.

Assume that a training set is given as $S={\left\{z_{i},y_{i}\right\}}_{i=1}^{l}$, Let *I*_+_ = {*i* | *y_i_* = +1}and *I*_−_ = {*i* | *y_i_* = −1}, then the ISVM is formulated as the constrained optimization problem:

(8) $$\begin{array}{l} \text{Minimize}\;\;\frac{1}{2}{\left|\left|w\right|\right|}^{2}+C^{+}\sum_{i\in I_{+}}\xi_{i}^{k}+C^{-}\sum_{i\in I_{-}}\xi_{i}^{k}\\ \text{subject}\;\text{to}\;\;\;y_{i}\left(w^{T}\varphi (x_{i})+b\right)-1+\xi_{i}\;\;\;\;i=1,\ldots ,l\\ \;\;\;\;\;\;\;\;\;\;\;\;\;\;\;\;\;\;\;\;\;\;\;\;\;\xi_{i}\geq 0 \end{array}$$

**1-Norm Soft Margin.** For *k* = 1 the primal Lagrangian is given by:

(9) $$L_{p}(w,b,\xi ,\alpha ,\beta )=\frac{1}{2}{\left|\left|w\right|\right|}^{2}+C^{+}\sum_{i\in I_{+}}\xi_{i}+C^{-}\sum_{i\in I_{-}}\xi_{i}-\sum_{i=1}^{l}\alpha_{i}\left(y_{i}\left(w^{T}\varphi (z_{i})+b\right)-1+\xi_{i}\right)-\sum_{i=1}^{l}\beta_{i}\xi_{i}$$

where *α_i_* and *β_i_* are Lagrange multipliers. By taking partial differential to the Lagrangian with respect to the primal variables, the dual problem becomes:

(10) $$\text{Maximize}\;\;L_{D}(\alpha )=\sum_{i=1}^{l}\alpha_{i}-\frac{1}{2}\sum_{i=1}^{l}\sum_{j=1}^{l}\alpha_{i}\alpha_{j}y_{i}y_{j}k(z_{i},z_{j})$$

subject to the constraints:

(11) $$\begin{array}{lll} 0\leq \alpha_{i}\leq C^{+} & \text{for} & y_{i}=+1\\ 0\leq \alpha_{i}\leq C^{-} & \text{for} & y_{i}=-1\\ \sum_{i=1}^{l}\alpha_{i}y_{i}=0 & & \end{array}$$

**2-Norm Soft Margin.** For *k*=2 we obatin the Lagrangian:

(12) $$L_{p}(w,b,\xi ,\alpha ,\beta )=\frac{1}{2}{\left|\left|w\right|\right|}^{2}+(C^{+}/2)\;\sum_{i\in I_{+}}\xi_{i}^{2}+(C^{-}/2)\sum_{i\in I_{-}}\xi_{i}^{2}-\sum_{i=1}^{l}\alpha_{i}\left(y_{i}\left(w^{T}\varphi (z_{i})+b\right)-1+\xi_{i}\right)-\sum_{i=1}^{l}\beta_{i}\xi_{i}$$

This results in a dual formulation as:

(13) $$L_{D}(\alpha )=\sum_{i=1}^{l}\alpha_{i}-\frac{1}{2}\sum_{i=1}^{l}\sum_{j=1}^{l}\alpha_{i}\alpha_{j}y_{i}y_{j}(k(z_{i},z_{j})+\prod_{\left[i\in I_{+}\right]}\frac{1}{C^{+}}\delta_{ij}+\prod_{\left[i\in I_{-}\right]}\frac{1}{C^{-}}\delta_{ij})$$

where Π_[·]_ is the indicator function. This can be viewed as a change in the Gram matrix *G*. Add 1/*C*^+^ to the elements of the diagonal of *G* corresponding to examples of positive class and 1/*C*^−^ to those corresponding to examples of the negative class:

(14) $${G^{\prime }}_{ii}=\{\begin{array}{lll} k(z_{i},z_{i})+\frac{1}{C^{+}} & \text{for} & y_{i}=+1\\ k(z_{i},z_{i})+\frac{1}{C^{-}} & \text{for} & y_{i}=-1 \end{array}$$

The Kuhn-Tucker (KT) complementary conditions are necessary for the optimality:

(15) $$\begin{array}{l} \alpha_{i}[y_{i}(w^{T}\varphi (z_{i})+b)-1+\xi_{i}]=0,\;i=1,2,\ldots ,l\\ \begin{array}{lll} \left(C^{+}-\alpha_{i}\right)\cdot \xi_{i}=0 & \text{for} & y_{i}=+1\\ \left(C^{-}-\alpha_{i}\right)\cdot \xi_{i}=0 & \text{for} & y_{i}=-1 \end{array} \end{array}$$

If 0 < *α_i_* ≤ *C*^+^ or 0< *α _i_* ≤ *C*^−^, the corresponding data points are called support vectors (SVs). The solution for the weight vector is given by

(16) $$w_{o}=\sum_{i=1}^{N_{s}}\alpha_{i}y_{i}\varphi (z_{i})$$

where *N*_s_ is the number of SVs. In the case of 0 < α*_i_* < *C*^+^ or 0 < α*_i_* < *C*^−^, we have ξ *_i_* = 0 according to the KT conditions. Hence, one can determine the optimal bias *b_o_* by taking any data point whose 0 < *α _i_* < *C*^+^ or 0 < *α _i_* < *C*^−^. Once the optimal pair (*w_o_*, *b_o_*) is determined, the class label for an unseen data *z* can be obtained by the decision function:

(17) $$D(z)=\text{sign}\left(\sum_{i=1}^{N_{s}}\alpha_{i}y_{i}k(z_{i},z)+b_{o}\right)$$

If *D*(*z*) = 1, then *z* belongs to the positive class; otherwise (*D*(*z*) = −1) it belongs to the negative class.

Since KPCA and ISVM are kernel learning machines, the kernel function needs to be determined first. The radial-basis function (RBF) is adopted as the kernel in this paper:

(18) $$k(z_{i},z_{j})=\text{exp}\left(-{\left|\left|z_{i}-z_{j}\right|\right|}^{2}/2\sigma^{2}\right)$$

where the width *σ* ^2^ is specified a *priori* by the user.

## 3. Experimental Section

According to the introduction to the inline inspection scheme in Section 2, we know that the performances of KPCA and ISVM dominate the inspection rate of the scheme. Therefore, in this section, we conduct several experiments to test the performances of the KPCA and the ISVM.

**Figure 5.**
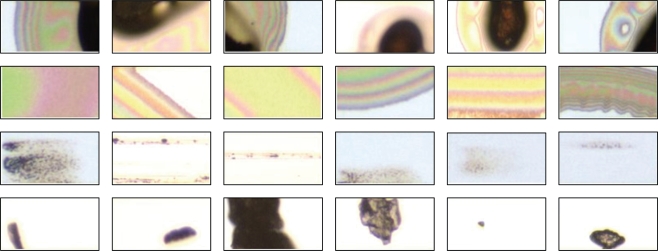
Defective PRs. The PRs in the top row are CGC PRs. The second and the third rows display some APC and SCR PRs, respectively. The PRs in the bottom row are PAR PRs.

A total of 540 defect images containing four kinds of inline defects (29 images for CGC, 18 images for APC, 52 images for SCR, 441 images for PAR) were used in the experiments. They were captured in a TFT array plant of a TFT-LCD manufacturer in Taiwan. After performing PR segmentation on each image, we obtained 930 defective PRs and 8,509 normal PRs in total. Since the normal PRs are almost the same in their appearances, we randomly selected 200 PRs from the 8,509 normal PRs as the experimental data. Among the 930 defective PRs, 88 PRs belong to CGC defect, 60 PRs belong to APC defect, 102 PRs belong to SCR defect, and 680 PRs belong to the PAR defect. We summarize the results in Table 2. Some defective PRs are displayed in Figure 5.

For KPCA, there are two parameters to be determined. One is the number of eigenvectors *d* and the other is the kernel parameter *σ*. Cross validation is an objective approach to parameter selection. The cross validation can be leave-one-out (LOO) [25], 2-fold [26], 5-fold [27], or 10-fold [24,25]. Considering that the available samples of some classes are not numerous, we use the 2-fold cross validation and the grid searching technique [5,24] to determine the optimal parameters and estimate the generalized classification accuracy of each method, because if using 5-fold cross validation, then only 12 APC PRs can be used for testing, which is obviously not enough. When using the 2-fold cross validation to search for the optimal parameters of KPCA, a simple nearest neighbor (NN) classifier is used as the classifier to measure the classification rate. The optimal parameters results in the best cross validation rate. Finally, the optimal pair of (*σ*,*d*) is found to be (3*e*4,127), which means that the dimensions of all input PRs are reduced substantially, from 2,500 to 127. In other words, 127 features will be extracted from each PR by KPCA. We also compare KPCA with PCA. The results are listed in Table 3. Note that the method “NN” means that the PR vectors are directly sent into the NN classifier without feature extraction.

From Table 3, it can be seen that when the PR vectors are directly sent into the NN classifier, the classification rate is only 84.07%. However, the classification rate can be improved to 89.02% when PCA is used as the feature extractor. Here the number of eigenvectors for PCA is 201, which is also determined by 2-fold cross validation. When KPCA is applied, the classification rate is further improved, from 89.02% to 92.03%. Clearly, using KPCA as the feature extractor can largely enhance the performance of the inline defect inspection. After using KPCA to extract features from all the PRs, each PR is represented by a vector of 127 × 1. Then, the 127-dimensional PR feature vectors are fed into the ISVM classifier for further defect classification.

In an ISVM, there are two different penalty weights, *C*^+^ and *C*^−^, while there is only one penalty weight in a SVM, *i.e.*, *C*. Obviously, training an ISVM would be more complicated. If the number of free parameters of ISVM can be reduced, the parameter selection in the training stage can be sped up. Recall that ISVM suggests that one should assign a larger penalty weight to the larger class, and a smaller one to the smaller class. Hence, we assume that the size of the positive training set and the size of the negative training set are *P* and *N*, respectively. Then the following equations can let us reduce the number of penalty weights from 2 to 1:

(19) $$\begin{array}{c} C^{+}=\frac{N}{P+N}\times C\\ C^{-}=\frac{P}{P+N}\times C \end{array}$$

where *C* is also a penalty weight. Clearly, once *C* is determined, both *C*^+^ and *C*^−^ are obtained automatically. Equations in (19) indicate that if *P* > *N*, then *C*^+^ < *C*^−^; if *P* < *N*, then *C*^+^ > *C*^−^. The advantage is that the number of free parameters of an ISVM is reduced from 3 (*C*^+^, *C*^−^, *σ*) to 2 (*C*, *σ*).

In the following, we compare ISVM with SVM. The one-against-one method and the voting strategy are also adopted to extend a binary SVM to multi-class SVMs. That is, since there are 5 classes, we need to train 10 SVMs in total. For fair comparison, the optimal parameters of the classifiers, including the ISVMs and the SVMs, are determined by using 2-fold cross validation. The best classification rates are reported in Table 4. Note that the 1N-ISVM and the 2N-ISVM in Table 4 denote the 1-norm and 2-norm soft margin ISVMs, respectively.

The results listed in Table 4 show that the classification rates of the NN, SVM, 1N-ISVM, and 2N-ISVM are 92.03%, 94.69%, 95.40%, and 96.28%, respectively. These results indicate that SVM performs better than the simple NN classifier. Also, ISVMs outperform SVM. It is not surprising, because from Table 2 we can see that the numbers of PRs in the five classes are different. That is, the training data sets are imbalanced. Thus, for the practical problem of LCD inline defect classification, ISVM classifier is more suitable than the regular SVM. Moreover, it can be seen from Table 4 that the proposed 2-norm soft margin ISVM is better than the 1-norm soft margin ISVM that has been used in the face recognition problem [23].

Several experiments are also conducted to compare different SVM methods without KPCA feature extraction. The results are listed in Table 4. As can be seen from Table 4, 2N-ISVM is still the best among the SVM methods. Moreover, the results illustrate the advantage of using KPCA: the methods (KPCA + SVM) (KPCA + 1N-ISVM), and (KPCA + 2N-ISVM) outperform SVM, 1N-ISVM, and 2N-ISVM by 1.57% (94.69-93.12), 1.17% (95.40%-94.23%), and 1.62% (96.28%-94.66%), respectively. The results indicate that when the SVM methods are used as the classifier, using the KPCA as the feature extractor can still improve the classification rates.

Table 4 shows the classification results. However, we also concern the defect detection result. Therefore, the detailed 2N-ISVM classification result of each class is listed in Table 5. For example, “Normal (100)” means that there are 100 normal PRs in total. Table 5 indicates that 1) one APC PR is classified as the normal class, 2) two PAR PRs are classified as the normal class, 3) one normal PR is classified as the PAR class and 4) for the two classes of CGC and SCR, no PRs are classified as the normal class. Therefore, the defect detection error rate is (1 + 2 + 1)/(44 + 30 + 51 + 340 + 100) = 0.71%. In other words, the detection rate is 99.29%. It is worth mentioning that the classification speed of ISVM is very fast, less than 0.1 seconds, because the decision function expressed in (17) depends only on the support vectors. In addition, it takes around 0.8 seconds to extract KPCA features from each PR. Hence, the defect inspection time for each PR is less than 1 second.

## 4. Conclusions

An inline defect inspection scheme for TFT-LCD array process has been presented in this paper. Because the defects of the TFT array process are various and are too small to be observed, we use high-resolution cameras to capture the images from the surfaces of the LCD panels. Then the proposed scheme is applied to detect and classify the defects in the images. In the proposed scheme, the KPCA technique is used to extract features from the images and the proposed 2-norm soft margin imbalanced SVM is used as the defect classifier. The effectiveness and the efficiency of the scheme have been demonstrated by the experimental results carried out on real LCD images.

The proposed inspection scheme is able to detect the defects within the PRs. However, if a defect happens only on GE or CS, the current scheme fails to detect it. Although such a situation seldom happens, it is still necessary to further develop a new inspection scheme that can detect the defects falling only on the GE and CS regions. That will be one of the future tasks.

## Figures and Tables

**Table 1.** Types of the inline defects in GE engineering and their causes.

**Inline Defects**	**Cause of generation**
CGC (Connection between GE and CS)	It involves particles left on the thin film before photoresist coating. It would make the connected GE line and CS becomes a short circuit after the etching process.
APC (Abnormal photo resist coating)	APC is caused by the staining of thinner drops on the thin film, which would produce drop-like photoresist holes. APC would make the pattern deformed after exposure.
SCR (Scratch)	1) when the robot arm for carrying glass substrates is biased in its position, substrates would get scratched; 2) when the cassette placing substrates deforms, substrates get scratched.
PAR (Particle)	When the panels are carried to the inspection equipment by RGV, particles would fall on the surfaces of the panels.

**Table 2.** Date set used in the experiments.

	**CGC**	**APC**	**SCR**	**PAR**	**Normal**	**Total**
# PRs	88	60	102	680	200	1130

**Table 3.** Comparison of classification rates (CR) among different feature extraction methods.

**Methods**	**NN**	**PCA + NN**	**KPCA + NN**
CR (%)	84.07	89.02	92.03

**Table 4.** Comparison of classification rates (CR) among different classification methods.

**Methods**	**KPCA + NN**	**KPCA + SVM**	**KPCA + 1N-ISVM**	**KPCA + 2N-ISVM**
CR (%)	92.03	94.69	95.40	96.28
**Methods**	**NN**	**SVM**	**1N-ISVM**	**2N-ISVM**
CR (%)	84.07	93.12	94.23	94.66

**Table 5.** 2N-ISVM classification results for each class.

**Classification results**	**CGC (44)**	**APC (30)**	**SCR (51)**	**PAR (340)**	**Normal (100)**

**CGC**	41	1	0	2	0
**APC**	2	28	0	0	1
**SCR**	0	0	48	8	0
**PAR**	1	0	3	328	0
**Normal**	0	1	0	2	99
